# Pediatric Pulmonary Arteriovenous Malformations in Patients with Hereditary Hemorrhagic Telangiectasia: Screening, Diagnosis, and Management

**DOI:** 10.3390/jcm14113739

**Published:** 2025-05-27

**Authors:** Claire S. Kaufman, Minh Anh Nguyen, Amy Bezold, Mark S. Chesnutt

**Affiliations:** 1 Pacific Northwest HHT Center of Excellence, Dotter Department of Interventional Radiology, Oregon Health and Science University, Portland, OR 97239, USA; chesnutm@ohsu.edu; 2 School of Medicine, Oregon Health and Sciences University, Portland, OR 97239, USA; nguyminh@ohsu.edu; 3 Dotter Department of Interventional Radiology, Oregon Health and Sciences University, Portland, OR 97239, USA; bezold@ohsu.edu

**Keywords:** hereditary hemorrhagic telangiectasia, pulmonary arteriovenous malformation, embolization, pulmonary arteriovenous fistula

## Abstract

Pulmonary arteriovenous malformations (PAVMs) are abnormal communications between a pulmonary artery and pulmonary vein that bypass the capillary bed, resulting in right-to-left shunting. The majority of PAVMs are associated with hereditary hemorrhagic telangiectasia (HHT), an autosomal dominant disease. Asymptomatic children with either a confirmed diagnosis of HHT or who are at risk of HHT from positive family history, as well as those with signs or symptoms concerning for HHT and/or PAVMs, should undergo screening for PAVMs at the time of clinical presentation or diagnosis. Screening in children can use a conservative approach (pulse oximetry, exercise intolerance testing, and chest radiograph) or transthoracic contrast echocardiography with agitated saline (TTCE). Pediatric patients with large or physiologically significant PAVMs should be treated with transcatheter embolization. Close follow-up is required after treatment to evaluate for interval growth of other PAVMs or reperfusion of the treated PAVMs.

## 1. Introduction

Pulmonary arteriovenous malformations (PAVMs) are abnormal communications between a pulmonary artery and pulmonary vein that bypass the capillary bed, resulting in right-to-left shunting [1]. Most pediatric PAVMs are congenital and associated with hereditary hemorrhagic telangiectasia (HHT); other causes include surgical correction of congenital heart disease (most notably after Glenn or Fontan procedures with reported rates of 25–60%), cirrhosis, trauma, Fanconi’s syndrome, mitral valve stenosis, and prior surgery [2,3,4].

While many patients are asymptomatic, PAVMs can present with complications or symptoms of the right-to-left shunt [5]. This can range from symptoms due to shunting such as dyspnea, exercise intolerance, clubbing, cyanosis, and hypoxemia to complications from paradoxical embolization (including but not limited to transient ischemic attacks (TIA), stroke, septic emboli, brain abscess, and myocardial infarction) or rupture [6,7].

HHT is an autosomal dominant disease associated with the abnormal development of communications between arteries and veins. Resulting clinical manifestations include epistaxis from nasal mucosal telangiectasia, cutaneous telangiectasias, cerebral arteriovenous malformations (AVMs), PAVMs, visceral AVMs (most commonly in the liver), and gastrointestinal tract bleeding [8]. The estimated prevalence of HHT in North America is 1:5000 [9]. HHT can be diagnosed clinically using the Curacao criteria or via positive genetic testing [10]. HHT is the result of mutations in the transforming growth factor beta (TGF-ß) pathway. Type 1 HHT occurs from mutations in the *endoglin* gene (ENG), while Type 2 HHT occurs from mutations in the *activin A receptor-type II-like* 1 gene (ACVRL1) [11,12]. *SMAD4* is known to cause the juvenile polyposis/HHT syndrome; however, it only accounts for 1–2% of cases of HHT [13]. Mutations in ENG and ACVRL1 have been shown to account for 96% of patients with HHT by clinical Curacao criteria [14].

Approximately 90% of patients with PAVMs will ultimately be diagnosed with HHT, while 30–50% of patients with HHT will develop PAVMs [15]. The prevalence of PAVMs in pediatric patients with HHT is close to that of adult populations; however, complications related to PAVMs are not as commonly reported [1,16]. Large pediatric cohort data have shown that PAVMs are more commonly seen in children with mutations in ENG, although PAVMs can be found across all genotypes [17]. Children with a family history of HHT or suspected HHT, therefore, require screening for the presence of PAVMs.

## 2. Screening

Asymptomatic children with either a confirmed diagnosis of HHT or who are at risk of HHT from positive family history, as well as those with signs or symptoms concerning for HHT and/or PAVMs, should undergo screening for PAVMs at the time of clinical presentation or diagnosis [18,19]. Repeat screening is recommended at least every five years in asymptomatic children with HHT, as small PAVMs may develop or enlarge over time [20]. PAVMs can have marked growth during puberty. One study reports a mean growth rate of 10% per year of the aneurysm sac on computed tomography (CT), which may indicate the need for shorter interval screening in certain pediatric patient populations [21].

Children who develop dyspnea, exercise intolerance or limitations, or other pulmonary symptoms should be assessed for hypoxemia and should be considered for other screening tests for PAVMs. While some experts advocate for transthoracic contrast echocardiography with agitated saline (TTCE) as the standard first-line test, others have employed a conservative non-invasive screening strategy to minimize testing burden during childhood while still identifying patients in need of treatment. A conservative screening strategy is favored by many because of the observation that, in children, the risk of complications from untreated PAVMs is low and is less than the procedural risks and possible long-term complications after transcatheter embolization from difficult to treat reperfusion [22].

### 2.1. Conservative Screening Approach

**Figure 1:**
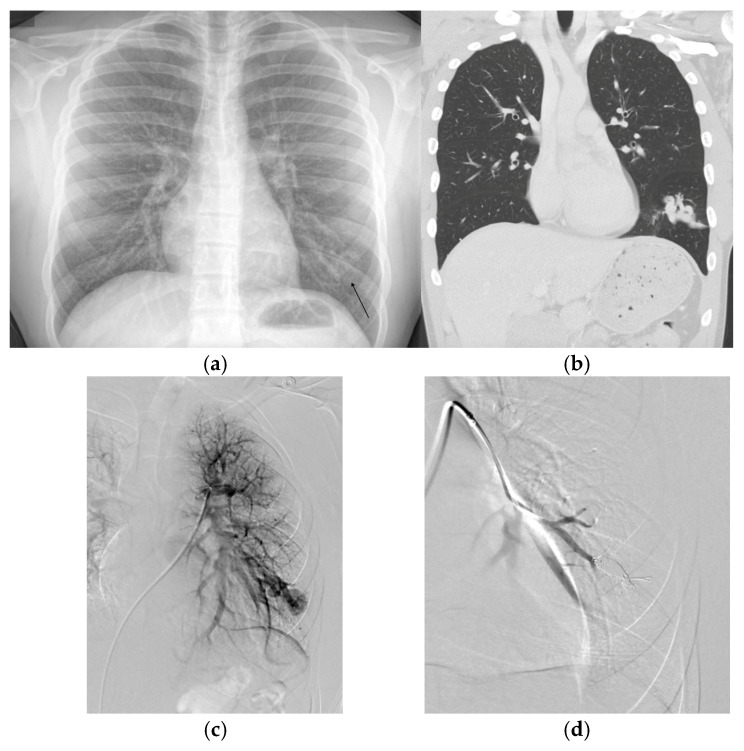
(**a**) Chest radiograph performed on an adolescent patient for shortness of breath and exercise intolerance demonstrating an opacity in the left lower lobe found to be a large pulmonary arteriovenous malformation (PAVM). The patient was subsequently diagnosed with hereditary hemorrhagic telangiectasia. (**b**) A subsequent chest computed tomography (CT) demonstrated the complex left lower lobe (LLL) PAVM. A non-contrast coronal image is displayed. (**c**) The corresponding pulmonary angiogram demonstrating the complex LLL PAVM. (**d**) Post embolization angiogram demonstrating successful occlusion.

A conservative non-invasive screening approach includes utilizing detailed clinical history, exercise intolerance testing or history, physical examination, pulse oximetry, and chest radiography (Figure 1). This approach has been shown to effectively identify clinically significant PAVMs requiring treatment in childhood, while avoiding the risks associated with IV placement, and intravenous injections in children for TTCE, and possible increased chance of more radiation exposure from a subsequent chest CT prompted by a positive TTCE [20,23]. This method has demonstrated safety over more than 7000 patient-years of follow-up with no reported PAVM-related complications in screened children [20]. However, there is concern that with the conservative screening protocol small PAVMs, that may lead to complications, may be missed until adulthood [24]. This has led to controversy in the literature and HHT community as to the preferred screening protocol, including recommendations from the Second International HHT Guidelines, include either chest radiograph and pulse oximetry or a TTCE for screening pediatric patients [18].

### 2.2. Transthoracic Contrast Echocardiography with Agitated Saline (TTCE)

TTCE (commonly referred to as “bubble echo”) is the gold standard screening test for PAVMs in adults and is also recommended as an option for screening children by the Second International HHT Guidelines [18]. TTCE has high sensitivity for detecting right-to-left shunts. During this screening, a transthoracic echocardiogram is performed with injection of agitated saline.

Despite the benefits of TTCE, in children, TTCE poses challenges including the need for intravenous access. Additionally, while sensitive, it may also detect smaller shunts unrelated to PAVMs but related to normal physiologic microvascular shunting, or shunts not amenable to endovascular treatment, leading to unnecessary follow-up imaging and radiation exposure with CT [23]. In a study of pediatric patients with a clinical diagnosis of HHT via Curacao criteria, patients with a low-grade echo bubble (Grade 0 or 1 Barzilai score) were found to be unlikely to have a treatable PAVM on chest CT and, therefore the study further recommended that follow-up with chest CT and imaging could be deferred in this clinical setting [25]. This is supported in the adult literature as well with the finding that only those patients with a grade 3 shunt using the quantitative score were found on chest CT imaging to have PAVMs amenable to embolization [26]. For these reasons, in some institutions, TTCE is postponed until adolescence or adulthood, favoring conservative screening protocols for younger patients [20].

### 2.3. Computed Tomography (CT)

Chest CT is not the first-line screening tool for patients with suspected PAVMs, but instead, this is the confirmatory imaging modality for patients with either a positive TTCE (shunt > grade 1), suspicious findings on chest X-ray, or abnormal clinical assessment. Chest CT is used to define PAVM size and morphology and to guide treatment planning [18]. In conservative screening approaches, CT is reserved for children with abnormal physical findings (e.g., cyanosis, digital clubbing), pulse oximetry <96%, or suspicious radiographic findings [20,23]. In children with known HHT and a positive TTCE of sufficient grade, CT is typically pursued regardless of symptoms [18]. Chest CT can be performed with or without contrast depending on institution protocol and preference. There are benefits to non-contrast chest CT imaging, especially in pediatric patients, such as avoiding risks from IV placement, which can be challenging in pediatric patients, paradoxical embolization from intravenous injections, contrast side effects, and the lower radiation dose without contrast [27].

## 3. Imaging Findings

### 3.1. Transthoracic Contrast Echocardiography with Agitated Saline (TTCE)

**Figure 2:**
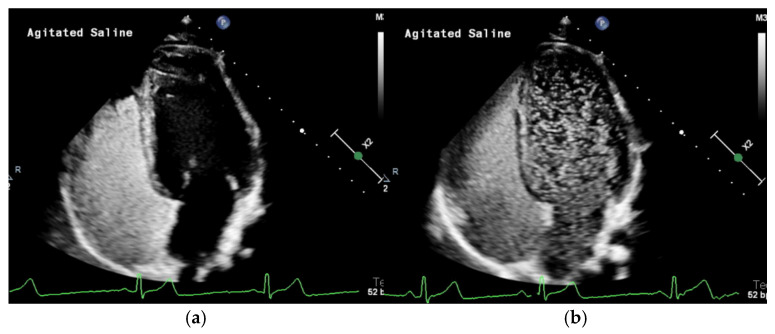
(**a**) Transthoracic contrast echocardiography with agitated saline (TTCE) demonstrates the opacification of the right heart with microbubbles. (**b**) TTCE in the same patient eight cardiac cycles later showing a grade 3 shunt from multiple large PAVMs.

As previously described, TTCE is the recommended initial imaging study for pediatric patients with suspected right-to-left shunting, including PAVMs. After standard transthoracic echocardiogram images are obtained, agitated saline contrast is injected peripherally during the sonographic four chamber imaging of the heart. This first shows the complete opacification of the right heart by microbubbles (Figure 2a). In patients without intracardiac or pulmonary shunts, these microbubbles will slowly dissipate out of the right heart and disappear, as they are trapped in the pulmonary capillary bed. However, in patients with a right-to-left shunt, microbubbles will appear in the left side of the heart (Figure 2b). This study is assessed based on the degree of delay in the appearance of microbubbles and the opacification of the left ventricular opacification after initial appearance in the right atrium. Intrapulmonary versus intracardiac shunting is differentiated based on the timing of the appearance of contrast in the left ventricle, measured in the duration of cardiac cycles. Intracardiac shunts such as a patent foramen ovale (PFO) microbubbles appear faster, usually within one to two cardiac cycles, whereas, with extracardiac or intrapulmonary shunts, microbubbles typically take longer to appear in the left heart, usually between three and eight cardiac cycles [26,28,29]. Intracardiac shunts such as a PFO may require Valsalva during TTCE to elicit shunting and is often performed as such during a second contrast injection [30]. Centers with expertise can sometimes identify the location of the shunt by draining bubbles from the specific pulmonary vein.

There are several systems commonly used to grade the degree of shunting on TTCE. The Brazilai score is based on the relative opacification of the ventricles by microbubbles. A grade 1 shunt demonstrates minimal opacification of the left ventricle with microbubbles, while the highest score, grade 4, demonstrates complete opacification with intense outlining of the left ventricle [26,31]. The quantitative score published by van Gent et al. counts the maximum number of microbubbles on each single frozen frame. Grade 1 has a maximum of 29 bubbles, grade 2 has 30–100 bubbles, and grade 3 has greater than 100 bubbles [26].

While TTCE is highly sensitive, false positives can occur. For example, an unroofed coronary sinus can appear with similar findings. In all cases with high-grade shunts (>grade 1), a chest CT should be performed for further evaluation.

### 3.2. Computed Tomography (CT)

**Figure 3:**
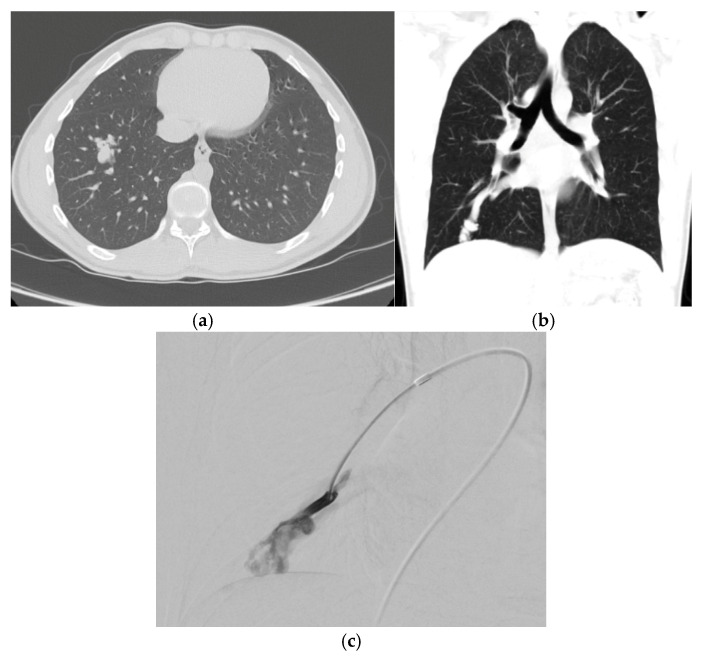
(**a**) Non-contrast axial computed tomography (CT) scan demonstrates a right lower lobe pulmonary arteriovenous malformation (PAVM) with clearly defined vessels but some residual surrounding ground glass in an adolescent patient with history of hypoxemia and transient ischemic attack. (**b**) Coronal non-contrast CT again demonstrating the large draining pulmonary vein with developing nidus. (**c**) Corresponding selective pulmonary angiogram demonstrates the developing nidus.

Multidetector CT (MDCT) imaging is the next step in the evaluation of patients with intrapulmonary shunting identified on TTCE, or concerns for PAVM using the conservative screening protocol. CT not only confirms a diagnosis of PAVMs, but is also used for treatment planning. A chest CT can be performed without contrast, using low dose protocols and thin slices (Figure 3). Maximum intensity projections (MIPs) can be reformatted to help identify PAVMs, and the afferent and efferent vasculature without adding additional scanning sequences [10,28]. Often, PAVMs are true pulmonary arteriovenous fistulas and are diagnosed by the identification of a feeding pulmonary artery and draining vein with an intervening aneurysmal sac. The characteristic feeding and draining pulmonary vessels can help differentiate PAVMs from other mimickers in the lungs, especially in the adult population. More recently, studies have examined the imaging findings of PAVMs in the pediatric patient population. Shin et al. proposed a grading system for pediatric PAVMs (Table 1) [32].

**Figure 4:**
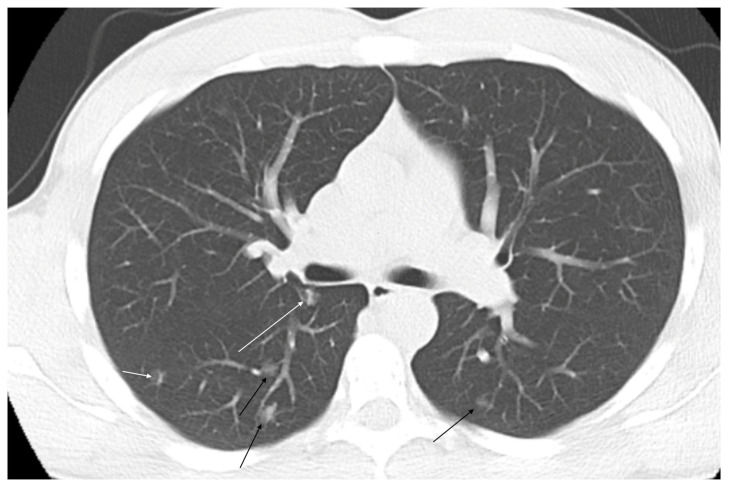
Multiple ground glass opacities in a pediatric hereditary hemorrhagic telangiectasia patient with hypoxemia and clubbing of the fingernails consistent with microvascular telangiectasias (black arrows). Linear vessels can be seen developing in several of the pulmonary arteriovenous malformations (white arrows).

CT can also identify probable microscopic telangiectasias, which form between arteriole and venules. Microscopic telangiectasias are visualized on CT as ground glass opacities [31]. As these opacities grow and mature over time, the vessels become apparent and the GGO are replaced first with identifiable vessels followed by an aneurysmal sac [28] (Figure 4). In general, as the PAVM matures, the pulmonary vein becomes the larger of the two vessels and can be traced back to a central pulmonary vein. Rarely, the arterial supply or draining vein can arise from a systemic source such as intercostal, phrenic, bronchial, or internal mammary arteries, although this variation becomes more common in cases of post transcatheter embolization reperfusion [33]. PAVMs have an increased prevalence in the lower lobes, middle lobe, and lingula [28].

PAVMs can be divided into simple, complex, telangiectatic, and diffuse types. A PAVM that is supplied by arterial feeders, one or more, from a single segmental pulmonary artery is classified as simple [34]. Complex PAVMs have more than one segmental feeding artery and represent approximately 10% of PAVMs [35]. Often, an intervening aneurysmal sac is identified.

More rare presentations of PAVMs can include telangiectatic or diffuse types. Diffuse PAVMs are defined as widespread AVMs involving most, or all, of a lobe or lung segment. These typically do not have a single focal aneurysmal dilation on the venous side. Instead, the vessels are more uniformly dilated and tortuous throughout the affected area [33]. Diffuse PAVMs may have simple or complex PAVMs within the segment. These are more difficult to treat given their diffuse nature [35].

Telangiectatic PAVMs were recently noted to be the fourth subtype of PAVM and have a ground glass appearance on chest CT. An associated vessel is not always identified; however, studies have found these to be associated with right-to-left shunting indicating the vascular nature of the lesion [36].

### 3.3. Angiography

MDCT has largely replaced angiography as the gold standard for the detection of PAVMs given its non-invasive nature and increased sensitivity [18]. However, angiography is still utilized as the first part of the embolization procedure, in cases of uncertain CT findings, and for diagnosis in cases of possible reperfusion. Pulmonary angiography is also used to identify reperfusion and the etiology of the reperfusion of previously treated PAVMs that may require further embolization given the limitations of MDCT in predicting reperfusion in some cases [6,37]. This can be performed from the main pulmonary artery but often also requires selective imaging performed in multiple projections utilizing specific protocols optimized for pulmonary angiography to provide the best visualization. Pulmonary angiography more accurately reveals the angioarchitecture of PAVMs, including the number and size of feeding arteries. It is not uncommon for an additional feeding pulmonary artery to be identified after embolization of an adjacent vessel.

## 4. Treatment

### 4.1. When to Treat

The treatment of pediatric patients with PAVMs is more nuanced and controversial than treatment of adults largely due to the lack of understanding of the natural history of pediatric PAVMs and limited data. The Second International HHT Guidelines recommend that pediatric patients with large PAVMs and PAVMs associated with decreased oxygen saturation undergo treatment with embolization [18]. This is further elaborated upon to specify that PAVMs with a feeding pulmonary artery of at least 3 mm are suitable for endovascular embolization [18]. PAVMs tend to grow during puberty with one study demonstrating a rate of increase in size of approximately 10% per year [21]. Therefore, PAVMs treated prior to puberty may be at an increased risk of reperfusion, and be more difficult to re-treat at a later date, from distorted angioarchitecture [38,39]. This is supported in the literature, with a reported incidence of reperfusion in pediatric patients of up to 70% and with some of these patients requiring more than one procedure to treat the reperfusion. This is compared to the reported reperfusion rates for adults of up to 50% [23,37,40,41]. A recent abstract found that there is a high percentage, 44%, of treated pediatric patients who developed systemic collateral blood supply. This, again, speaks to the potential downstream difficulties that may arise after the treatment of PAVMs in the pediatric population [36].

While many children with PAVMs are asymptomatic with lesions detected upon screening, children have also presented with severe complications from PAVMs including rupture, cyanosis, stroke, and other paradoxical embolization including myocardial ischemia [42]. The embolization of pediatric PAVMs is safe and effective and, therefore, should be performed in patients at risk of complications [39]. Given the higher risk of reperfusion due to the growth of the PAVM, which can be difficult to treat later in life, small asymptomatic PAVMs should be monitored and treated after puberty. This is supported by a recent case series that found a 23.1% rate of persistent in treated pediatric PAVMs aged less than 15 at the time of treatment, which was found to have a significant association with PAVM persistence [22]. Screening and continued surveillance after PAVM diagnosis or treatment is crucial in the pediatric population due to the higher rate of growth and appearance of PAVMs not previously identified [21,42].

### 4.2. How to Treat

#### 4.2.1. Embolotherapy

Transcatheter embolization remains the gold standard for the treatment of the majority of PAVMs and has been shown to be safe and effective in pediatric patients [39]. This procedure should be performed with general anesthesia for younger pediatric patients; however, moderate sedation can be considered for older patients. Once pulmonary artery access is obtained via femoral vein puncture, a long flexible guide sheath should be placed for stability. Depending on the patient’s size, this may be the same sheath that is used in the adult population or smaller. Drip lines should be attached to all sheaths, and care should be taken not to introduce air bubbles into the system to mitigate the risk of paradoxical embolization. The patient should be heparinized with weight-based heparin (50–100 u/kg), followed by activated clotting time (ACT) with a goal of 200, similar to the recommended goal for pediatric cardiac catheterization [43,44]. Angiographic injection rates and volumes should be tailored to the size of the patient and decreased from that used in the adult population, if indicated. All medications including lidocaine, antibiotics, and contrast need to be given in weight-based dosing [43] (Table 2). In cases of small children, care needs to be taken with flushing catheters due to possible intravascular hemodilution from saline [43]. Embolization should otherwise be performed in standard fashion using the appropriate mechanical embolic agent, coils, or plugs. It is crucial the interventionalists performing pediatric PAVM embolization be comfortable and experienced with pulmonary angiography and the nuances of embolizing PAVMs, as well as treating pediatric patients, to help achieve not only immediate technical success, but the best chance of durable treatment given the high risks of reperfusion.

#### 4.2.2. Surgery

While there has been a large movement away from surgical intervention for PAVMs, surgery may still play a role in specific patient populations. Diffuse PAVMs, while uncommon with a reported incidence of 4%, have higher morbidity and mortality. These lesions can be very difficult to treat endovascularly [45,46,47]. Embolization is not possible at the level of the AVM nidus and must be performed proximally due to the diffuse small vessel communication characteristic of these lesions. This can lead to complications over time including hemoptysis, hypoxemia, and the further recruitment of adjacent pulmonary or bronchial vessels [48]. A recent publication reported that, after surgical resection, segmentectomy, or lobectomy, in 11 pediatric patients with diffuse pulmonary AVMs experienced increased oxygen saturation and a decrease in clinical symptoms. This study suggests that this may be an alternative treatment option in this challenging patient population [47].

## 5. Follow-Up Post Treatment

Follow-up with CT and/or TTCE is crucial after embolization of pediatric PAVMs. This is supported in the Second International HHT Guidelines; however, the routine interval is not specified but, instead, left to center discretion [18]. Initial follow-up imaging is recommended 6–12 months after embolization [24]. Historically, this has been with chest CT; however, more recent studies in the adult literature support the use of graded TTCE for initial follow-up, reserving chest CT for those with more than a low-grade shunt [49,50]. It is crucial to continue screening after the initial post-procedure imaging. If there is no evidence of reperfusion or other new/enlarging PAVMs that need treatment the re-screening, interval can be increased to every 3 years [10]. Chest CT imaging can be performed with or without intravenous contrast depending on institutional protocols. We favor without contrast for our standard follow-up chest CT given the risks of additional IV injections, lower radiation dose without contrast, and limited added information due to the inherent differences in Hounsfield units providing good contrast between aerated lung and the vasculature.

## 6. Conclusions

Pediatric patients with HHT have a high prevalence of pulmonary arteriovenous malformations. Screening either with pulse oximetry and chest radiograph, or with TTCE is crucial for all pediatric patients with known or suspected HHT. The treatment of PAVMs that are large and physiologically significant or have been associated with complications should be performed in concordance with the Second International HHT Guidelines. This most often is via pulmonary angiography and transcatheter embolization, although procedural modifications due to patient size and weight may be necessary. Due to the PAVM immaturity and growth of PAVMs over time, pediatric patients treated with transcatheter embolization are at an increased risk of reperfusion, leading to lesions that are difficult to retreat or complications. Pediatric PAVM patients require close attention and follow-up. To provide the best care to this population, providers must be aware of the nuances in the care and management of pediatric HHT patients with PAVMs.

## Figures and Tables

**Table 1:** Multi-detector computed tomography imaging grading of pediatric PAVMs.

Grade	Findings
0	Nodule, unlikely to be a PAVM
1	GGO (telangiectasia of the post capillary venules and perivascular inflammation)
2	GGO with increased intralesional vascularity
3A	GGO with identifiable afferent artery located in the center of the opacity
3B	GGO increasingly replaced by the distinguishable vascular components and a single afferent artery
4A	GGO or nodule with two or more identifiable vessels (afferent artery and efferent vein) with the efferent vein located at the periphery
4B	GGO or nodule with two or more identifiable vessels (afferent artery and efferent vein); the vascular component replaces the nodule
5	Nodule with efferent vein larger than the afferent artery regardless of number of vessels without mature sac
6	Mature PAVM with enlarged sac, identifiable afferent artery and efferent vein

GGO—ground glass opacities, PAVM—pulmonary arteriovenous malformation.

**Table 2:** Pediatric medication dosing for angiography.

Medication	Dose
Lidocaine without epinephrine	5 mg/kg subcutaneous
Lidocaine with epinephrine	7 mg/kg subcutaneous
Heparin	50–100 u/kg IV, initial bolus
Bupivacaine 0.25%	2.5 mg/kg subcutaneous
Contrast medium	6–8 mL/kg IV
Cefazolin	25–50 mg/kg/dose (max 2 g)

IV—intravenous, mg—milligram, kg—kilogram, u—units, mL—milliliter.

## Data Availability

No new data were created or analyzed in this study.

## Abbreviations

The following abbreviations are used in this manuscript:

PAVM	Pulmonary arteriovenous malformation
HHT	Hereditary hemorrhagic telangiectasia
AVM	Arteriovenous malformation
TIA	Transient ischemic attacks
TTCE	Transthoracic Contrast Echocardiography with Agitated
GGO	Ground glass opacities
CT	Computed tomography
ACT	Activated clotting time
